# The Slow-Releasing and Mitochondria-Targeted Hydrogen Sulfide (H_2_S) Delivery Molecule AP39 Induces Brain Tolerance to Ischemia

**DOI:** 10.3390/ijms22157816

**Published:** 2021-07-22

**Authors:** Bartosz Pomierny, Weronika Krzyżanowska, Jakub Jurczyk, Alicja Skórkowska, Beata Strach, Małgorzata Szafarz, Katarzyna Przejczowska-Pomierny, Roberta Torregrossa, Matthew Whiteman, Monika Marcinkowska, Joanna Pera, Bogusława Budziszewska

**Affiliations:** 1 Department of Toxicological Biochemistry, Faculty of Pharmacy, Jagiellonian University Medical College, Medyczna 9, 30-688 Kraków, Poland; weronika.krzyzanowska@uj.edu.pl (W.K.); jakub.jurczyk@doctoral.uj.edu.pl (J.J.); alicja.skorkowska@doctoral.uj.edu.pl (A.S.); boguslawa.budziszewska@uj.edu.pl (B.B.); 2 Department of Neurology, Faculty of Medicine, Jagiellonian University Medical College, Botaniczna 3, 31-503 Kraków, Poland; beata.strach@doctoral.uj.edu.pl (B.S.); joanna.pera@uj.edu.pl (J.P.); 3 Department of Pharmacokinetics and Physical Pharmacy, Faculty of Pharmacy, Jagiellonian University Medical College, Medyczna 9, 30-688 Kraków, Poland; malgorzata.szafarz@uj.edu.pl (M.S.); katarzyna.przejczowska@uj.edu.pl (K.P.-P.); 4 St. Luke’s Campus, University of Exeter Medical School, Exeter EX1 2LU, UK; r.torregrossa@ex.ac.uk (R.T.); M.Whiteman@exeter.ac.uk (M.W.); 5 Department of Medicinal Chemistry, Faculty of Pharmacy, Jagiellonian University Medical College, Medyczna 9, 30-688 Kraków, Poland; monika.marcinkowska@uj.edu.pl

**Keywords:** brain ischemia, hydrogen sulfide, AP39, brain preconditioning, neuroinflammation, mitochondrial targeting

## Abstract

Ischemic stroke is the third leading cause of death in the world, which accounts for almost 12% of the total deaths worldwide. Despite decades of research, the available and effective pharmacotherapy is limited. Some evidence underlines the beneficial properties of hydrogen sulfide (H_2_S) donors, such as NaSH, in an animal model of brain ischemia and in in vitro research; however, these data are ambiguous. This study was undertaken to verify the neuroprotective activity of AP39, a slow-releasing mitochondria-targeted H_2_S delivery molecule. We administered AP39 for 7 days prior to ischemia onset, and the potential to induce brain tolerance to ischemia was verified. To do this, we used the rat model of 90-min middle cerebral artery occlusion (MCAO) and used LC-MS/MS, RT-PCR, Luminex^TM^ assays, Western blot and immunofluorescent double-staining to determine the absolute H_2_S levels, inflammatory markers, neurotrophic factor signaling pathways and apoptosis marker in the ipsilateral frontal cortex, hippocampus and in the dorsal striatum 24 h after ischemia onset. AP39 (50 nmol/kg) reduced the infarct volume, neurological deficit and reduced the microglia marker (Iba1) expression. AP39 also exerted prominent anti-inflammatory activity in reducing the release of Il-1β, Il-6 and TNFα in brain areas particularly affected by ischemia. Furthermore, AP39 enhanced the pro-survival pathways of neurotrophic factors BDNF-TrkB and NGF-TrkA and reduced the proapoptotic proNGF-p75^NTR^-sortilin pathway activity. These changes corresponded with reduced levels of cleaved caspase 3. Altogether, AP39 treatment induced adaptative changes within the brain and, by that, developed brain tolerance to ischemia.

## 1. Introduction

Stroke is one of the major causes of death and disabilities in adults worldwide. There are 15 million new cases of brain ischemia every year, and it is estimated that this is the most frequent cause of long-term disabilities and permanent invalidities among adults [1]. Unfortunately, there is no effective treatment applicable for all stroke patients. The intravenous administration of a recombinant tissue plasminogen activator (rt-PA) is the only available pharmacotherapy but cannot be used in all patients [2]. The development of additional therapeutic procedures is hampered by insufficient knowledge of the molecular mechanism involved during brain ischemia and in the reperfusion phase after restoration of the blood supply. As such, there is a great social need to develop new pharmacotherapy strategies that would allow us to save many lives.

Up until recently, hydrogen sulfide (H_2_S) was seen as a toxic gas. Indeed, high doses (above 50 µM) of H_2_S exert a neurotoxic effect because of the inhibition of cytochrome c oxidase in mitochondrial respiration. H_2_S is a small molecule that is, together with NO and CO, termed a gasotransmitter. These molecules play an important role in numerous physiological processes [3,4]. It is known that H_2_S acts as an intracellular messenger and neuromodulator [5]. H_2_S is produced endogenously at a generally low concentration, and three main enzymes: cystathionine-β-synthase, cystathionine-γ-lyase and 3-merkaptopyruvate sulfotransferase are involved in its synthesis [6,7]. It is an important compound in cellular energy metabolism in the peripheral tissues and in the brain [8]. H_2_S signaling targets the mitochondria on many levels, affecting mitochondrial respiration, as well as mitochondrial-controlled apoptosis. H_2_S modulates the activity of the electron transport chain of the mitochondria in a concentration-dependent manner. The current dogma states that, at low concentrations, H_2_S acts as electron donor to the electron transport chain via its own irreversible oxidation by sulfide-quinone oxidoreductase (SQOR, low expression in the central nervous system; CNS). The electrons obtained from H_2_S oxidation enter the electron transport chain through complex III. This activity intensifies oxidative phosphorylation and ATP synthesis [9,10,11]. As such, enhanced H_2_S metabolism may protect the mitochondria during ischemia or oxygen and glucose deprivation. On the other hand, H_2_S at high concentrations above 50 µM inhibits the electron transport chain, leading to redox imbalance, cellular proliferation inhibition and metabolism shifting toward reductive carboxylation [12].

The current evidence indicates that H_2_S plays an important role in CNS signaling, acting as an intracellular messenger and neuromodulator [5]. Through the use of H_2_S-generating compounds such as NaSH, H_2_S has been shown to modulate NMDA receptors and upregulate GABA-B receptors [13,14]. Data shows that H_2_S protects neurons from oxidative stress, demonstrating similar properties to the most potent brain antioxidant, glutathione (GSH), and what is more, it stimulates GSH production [15,16]. It has been shown that H_2_S suppresses oxidative stress, scavenging reactive oxygen species (ROS) by acting directly within the mitochondria, where ROS are produced [17,18]. H_2_S raises the astroglial and microglial intracellular calcium levels and, thus, modulates their activity. It has been shown that H_2_S has an anti-inflammatory effect through reducing the release of proinflammatory cytokines and increasing the level of anti-inflammatory cytokines [19,20,21]. Such effects of H_2_S were also observed in neurological disorders, including neurodegenerative diseases like Alzheimer’s disease or Parkinson’s disease [22,23], suggesting that H_2_S-generating compounds may be effective in counteracting these detrimental effects. The pathophysiological role of H_2_S has been studied in various diseases, including cardiovascular diseases and hematologic diseases but, first, in neurological diseases. Nevertheless, the role of H_2_S in cerebral ischemia is still unclear. There are data from both animal as well as in vitro studies confirming the neuroprotective effect of an inorganic H_2_S donor, NaSH in ischemia/reperfusion (I/R) or oxygen glucose deprivation (OGD) models [24,25]. Relatively low doses of H_2_S donors, predominantly NaSH (i.e., 25-µmol/kg b.w. i.v.), exert a neuroprotective effect after cerebral ischemia and in animal models of hypoxia [26,27,28,29]. NaSH (at doses of 0.2 and 0.4 µmol/kg) administered to animals 30 min after ischemia onset has an anti-inflammatory, antiapoptotic and antioxidative effect [30]. NaSH (at the dose of 60 µmol/kg) administered at the beginning of a 60-min middle cerebral artery occlusion (MCAO) significantly reduced the infarct size, in addition to decreasing the blood–brain barrier damage [31]. H_2_S donors used as a preconditioning strategy also reveal a beneficial effect in I/R injury of the retina, liver or intestine, but there are no data on inducing brain tolerance to ischemia [32,33,34,35,36]. On the other hand, there are reports showing the neurotoxic effects of H_2_S donors or ameliorating effect of H_2_S scavengers or inhibitors of enzymes synthesizing H_2_S in vivo [37,38]. Inorganic sulfide salts (Na_2_S and NaSH) have been predominantly employed to study the H_2_S effect on brain ischemia. However, there is a great concern that inorganic H_2_S donors generate H_2_S instantaneously at a high concentration and, thus, may evoke a neurotoxic effect. The neuroprotective activity of the generated H_2_S is dose-dependent, and only a relatively low concentration below 50 µM, using stable H_2_S donors, may yield the desired protection after ischemia. For this reason, inorganic compounds are unreliable sources of H_2_S and may lead to misinterpretation of the obtained results [16].

One of the promising compounds is AP39, a novel, stable and mitochondria-targeted H_2_S donor. AP39 [(10-oxo-10-(4-(3-thioxo-3H-1,2-dithiol-5yl) phenoxy) decyl) triphenyl phosphonium bromide] consists of a mitochondria-targeting motif, triphenylphosphonium (TPP+), coupled to a H_2_S-donating moiety (dithiolethione) by an aliphatic linker [39]. As a result of such a chemical structure, AP39 releases H_2_S within the intramembrane space of the mitochondria. The dysfunction of these organelles, occurring after brain ischemia, is probably the first step of the pathologic events that lead inter alia to a cellular energetic imbalance, oxidative stress, neuroinflammation and, eventually, the initiation of various mechanisms of cell death. Spatiotemporally, these processes correlate with a decrease of the H_2_S level in the brain tissue and the disrupted homeostasis of this gaseous neurotransmitter after cerebral ischemia [29]. 

In this research, the potential neuroprotective effect of AP39 was analyzed. This study was undertaken to determine whether mitochondrial H_2_S induces adaptative changes in the brain and, thus, induces brain tolerance to ischemia. This phenomenon, called brain preconditioning, involves induction in the brain of a tolerance to strong, damaging stimuli such as ischemia. The low intensity of potentially damaging stimuli, such as short-term ischemia or a subtoxic dose of 3-nitropropionic acid (3NP), produce such effects on the brain [40]. Since H_2_S, similarly to 3NP, at toxic concentrations disrupts the mitochondrial respiratory chain, low doses of the H_2_S donor AP39, in the same way as 3NP, may potentially serve as brain preconditioning mediator. In this study, animals were exposed to seven low-concentration doses of AP39, and 3 days after the last dose, the animals were subjected to surgical procedure of 90-min MCAO or SHAM. Such a dosage scheme is used in preconditioning strategies to induce molecular adaptative changes, which orchestrate the mechanism of brain tolerance to ischemia [41,42,43]. The lag time of three day is to exclude the possible effects of the last dose. Twenty-four hours after reperfusion, the neurological deficit was assessed; next, the animals were sacrificed, and the obtained tissues were analyzed for the infarct volume and the level of free and bounded H_2_S but also for the effect of the treatment on the process of neuroinflammation, level of neurotrophic factors and their signaling pathways and the marker of apoptosis. In the performed assays, three ipsilateral brain structures were analyzed: the dorsal striatum, the frontal cortex and the hippocampus. These brain structures represent areas that are more or less affected by brain ischemia [44,45,46].

## 2. Results

### 2.1. AP39 Treatment Reduced the Neurological Deficit and Infarct Volume

**Figure 1:**
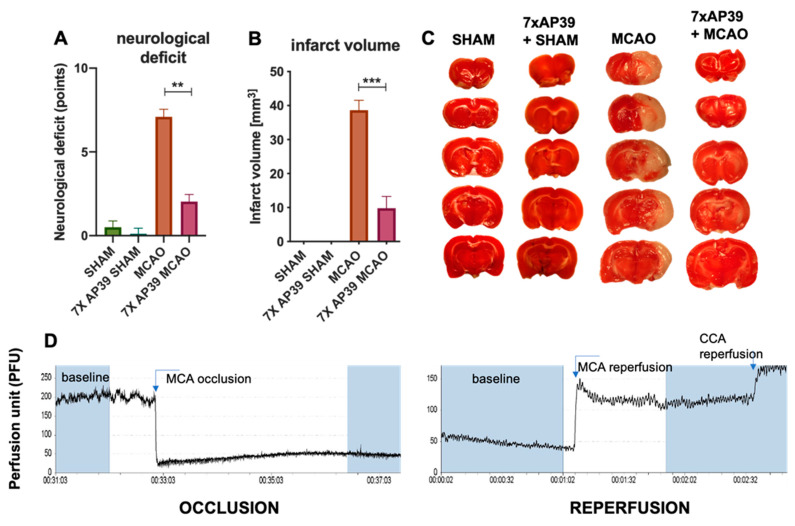
The effect of AP39 on the neurological deficit and the infarct volume. (**A**) Neurological deficit determined by Philips’s scale, *n* = 8, Mann–Whitney test, ** *p* < 0.01 vs. MCAO. (**B**) Infarct volume, *n* = 6, Student’s *t*-test, *** *p* < 0.001 vs. MCAO. (**C**) Representative photographs of TTC staining of the brain sections for determination of the infarct volume. Data are presented as the mean ± SEM. (**D**) An exemplary blood flow graph recorded separately during MCA occlusion (76% blood flow reduction) and MCA, CCA reperfusion. MCA—middle cerebral artery and CCA—common carotid artery.

The procedure of 90-min MCAO resulted in a significant infarction after 24 h of reperfusion. An average score of the neurological deficit for the MCAO group was 7.097 ± 2.495. Within contrast, in the AP39 group, there was significantly reduced neurological deficit score (2.042 ± 2.074; *p* = 0.0005) at 24 h postreperfusion (Figure 1A). TTC staining procedure revealed that the 7-day treatment with AP39 reduced the infarct volume by 74.6% when compared with the MCAO group (38.65 ± 2.898 mm^3^ MCAO vs. 9.815 ± 3.444 mm^3^ 7xAP39 MCAO, *p* < 0.0001). The SHAM-operated animals did not show any significant changes in their neurological deficits and infarctions (Figure 1B,C).

### 2.2. AP39 Prevented the MCAO-Induced Depletion of Free and DTT-Releasable H_2_S Levels

**Figure 2:**
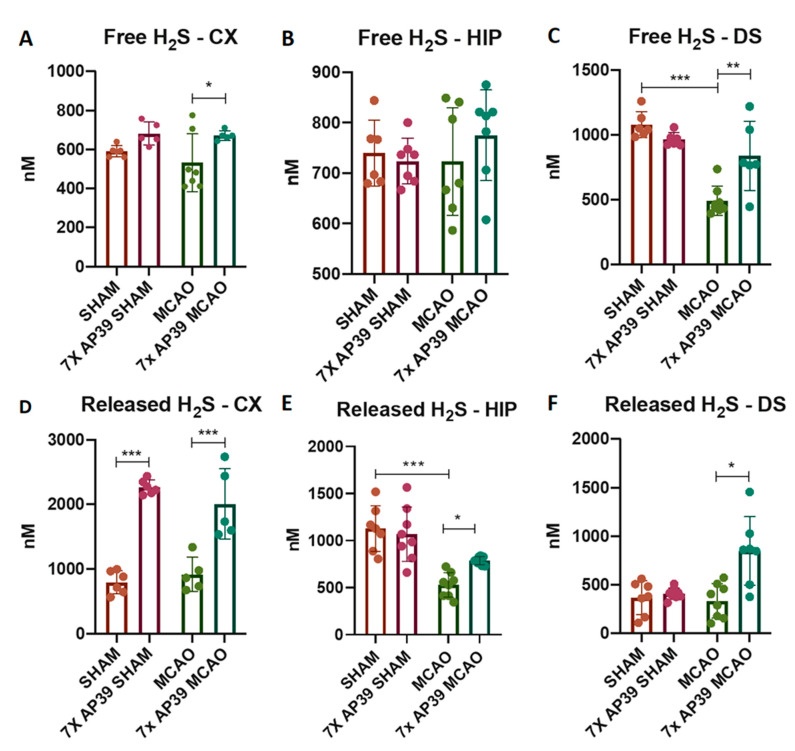
The effect of MCAO and AP39 on the levels of (**A**–**C**) free H_2_S and (**D**–**F**) DTT-released H_2_S in the frontal cortex (CX), hippocampus (HIP) and in the dorsal striatum (DS), *n* = 8, one-way ANOVA, followed by Sidak’s correction for post hoc comparisons; * *p* < 0.05, ** *p* < 0.01 and *** *p* < 0.001 vs. MCAO or SHAM. Data are presented as the mean ± SD.

MCAO significantly reduced the level of free H_2_S in the dorsal striatum by 54.42%, when compared with the SHAM animals, with no such effect in the frontal cortex or in the hippocampus. In the animals subjected to MCAO, the AP39 treatment significantly raised the free H_2_S in the frontal cortex and in the dorsal striatum by 26.36% and 70.47%, respectively (*p* = 0.0431 and *p* = 0.001, respectively), compared to the MCAO animals (Figure 2). 

Since it has been proposed that H_2_S exists in vivo in a free form but, also, in reversible bound forms such as sulfanes, polysulfides and persulfides, we indirectly quantified these forms by digesting tissue homogenates with DTT. As such, we determined the concentration of released H_2_S. Interestingly, in the MCAO group (compared with the SHAM group), we observed significant reduction of released H_2_S only in the hippocampus (by 53.14%, *p* = 0.0001), with no significant effects in the other brain structures. In the 7xAP39 MCAO group, a significant increase was observed in the frontal cortex and in the hippocampus and, also, in the dorsal striatum (by 118.95%, 48.81% and 155.01%, *p* < 0.0001, *p* = 0.045 and *p* = 0.0002, respectively) compared to the MCAO group. Comparing the experimental group treated with AP39 subjected to the SHAM procedure with the SHAM animals pretreated with the vehicle, a significant increase of released H_2_S was observed in the frontal cortex (185.6%, *p* < 0.0001) but not in the other brain structures (Figure 2). 

### 2.3. AP39 Mitigated the Neuroinflammation after MCAO

Il-1ß, Il-6 and TNFα are proinflammatory cytokines engaged in the initiation and progression of neuroinflammation. This process is one of the hallmarks of lesion formation in the natural course of brain ischemia [47]. Il-10 is an anti-inflammatory cytokine known to promote neuroprotection and mitigate the cytotoxic effects of neuroinflammation occurring after brain ischemia [48]. The levels of Il-1ß, Il-6, Il-10 and TNFα and mRNA encoding these cytokines were measured using the Luminex^TM^ and RT-qPCR techniques, respectively. The neuroinflammation process is mainly orchestrated by microglia, specifically its activation and polarization towards cytotoxic M1-like cells and cytoprotective M2-like cells. To assess these processes, the expression level of the microglia marker (Iba1), M1-like microglia phenotype marker (CD86) and M2-like microglia phenotype marker (CD206) were determined using an immunoblot.

**Figure 3:**
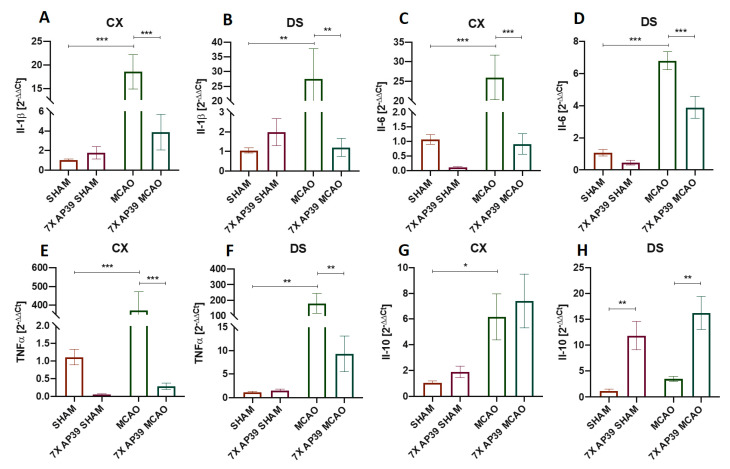
The effect of MCAO and AP39 on the mRNA expression level of the proinflammatory and anti-inflammatory cytokines in the frontal cortex (CX) and in the dorsal striatum (DS). (**A**,**B**) Relative Il-1ß mRNA levels in the CX and DS, respectively. (**C**,**D**) Relative Il-6 mRNA levels in the CX and DS, respectively. (**E**,**F**) Relative TNF-α mRNA levels in the CX and DS, respectively. (**G**,**H**) Relative Il-10 mRNA levels in the CX and DS, respectively. *n* = 8, one-way ANOVA, followed by Sidak’s correction for post hoc comparisons; * *p* < 0.05, ** *p* < 0.01 and *** *p* < 0.001 vs. MCAO or SHAM. Data are presented as the mean ± SEM.

The expression levels of the specific mRNA encoding Il-1ß, Il-6, Il-10 and TNF-α were determined using RT-qPCR assays. MCAO resulted in a significant increase of mRNA for Il-1ß, Il-6 and TNFα in the frontal cortex and in the dorsal striatum (Figure 3). Comparing MCAO and 7xAP39 MCAO revealed that the treatments significantly reduced the mRNA expression levels of the proinflammatory cytokines in the frontal cortex, as well as in the dorsal striatum. The mRNA expression level of Il-10 was significantly elevated after the MCAO procedure in the frontal cortex but not in the dorsal striatum. The treatment with AP39 resulted in a significantly increased Il-10 mRNA expression in the dorsal striatum in both experimental groups: 7xAP39 SHAM (vs. SHAM, *p* = 0.0059) and 7xAP39 MCAO (vs. MCAO, *p* = 0.0012); however, it did not elicit such an effect in the frontal cortex (Figure 3). Neither ischemia nor the AP39 treatment elicited any changes in the hippocampus (data not shown).

**Figure 4:**
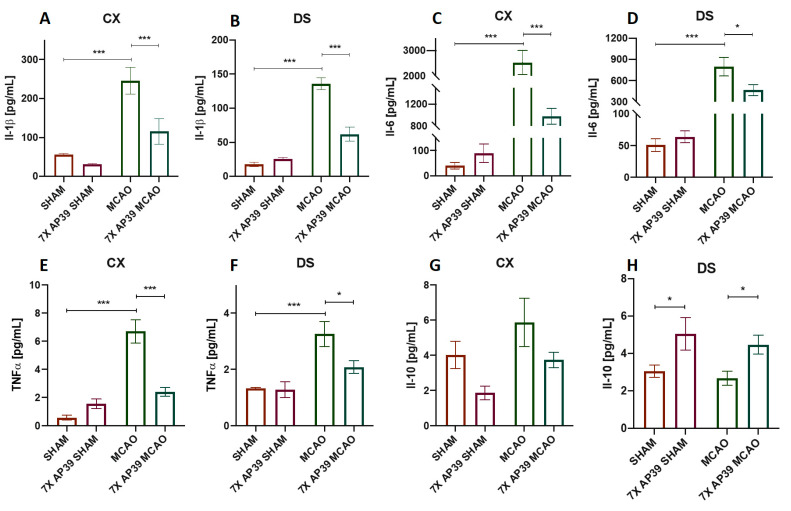
The effect of MCAO and AP39 on the protein expression level of the proinflammatory and anti-inflammatory cytokines in the frontal cortex (CX) and in the dorsal striatum (DS). (**A**,**B**) Relative Il-1β levels in the CX and DS, respectively. (**C**,**D**) Relative Il-6 levels in the CX and DS, respectively. (**E**,**F**) Relative TNFα levels in the CX and DS, respectively. (**G**,**H**) Relative Il-10 levels in the CX and DS, respectively. *n* = 8, one-way ANOVA, followed by Sidak’s correction for post hoc comparisons, * *p* < 0.05 and *** *p* < 0.001 vs. MCAO or SHAM. Data are presented as the mean ± SEM.

MCAO resulted in a significant increase of the proinflammatory cytokine levels, such as Il-1ß, Il-6 and TNFα in the frontal cortex, as well as in the dorsal striatum. In animals receiving AP39 and subjected to MCAO (7xAP39 MCAO), a significant reduction was observed for Il-1ß, Il-6 and TNF-α when compared with the MCAO group. AP39 increased the concentration of Il-10 in both experimental groups 7xAP39 SHAM (vs. SHAM, *p* = 0.0355) and 7xAP39 MCAO (vs. MCAO, *p* = 0.037) in the dorsal striatum; however, this effect was not observed in the frontal cortex (Figure 4). There were no changes with these parameters between the groups in the hippocampus (data not shown).

**Figure 5:**
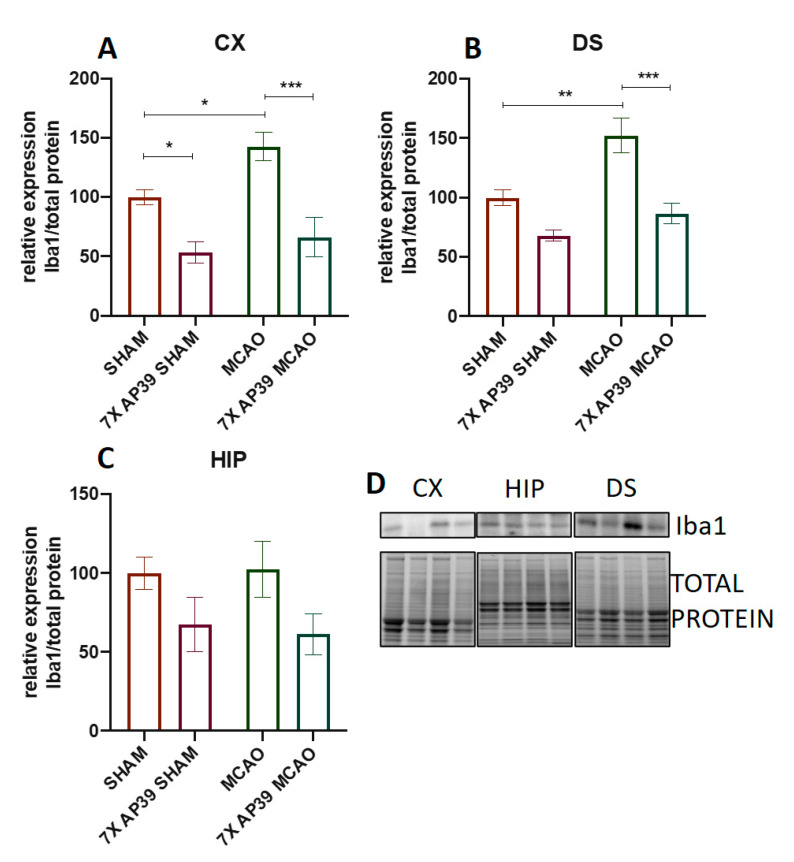
The influence of MCAO and AP39 on the protein expression level of Iba1 in (**A**) the frontal cortex (CX), (**B**) dorsal striatum (DS) and (**C**) in the hippocampus (HIP). (**D**) Immunoblots representative for the CX, HIP and DS for Iba1, together with the total protein amount visualized by the stain-free technique. The order of the samples is the same as in the graphs. *n* = 6, one-way ANOVA, followed by Sidak’s correction for post hoc comparisons; * *p* < 0.05, ** *p* < 0.01 and *** *p* < 0.001 vs. MCAO or SHAM. Data are presented as the mean ± SEM.

**Figure 6:**
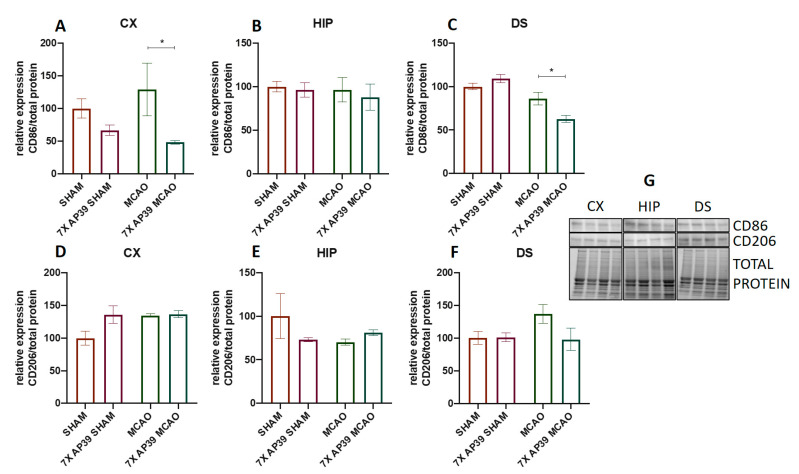
The effect of MCAO and AP39 on the protein expression of (**A**–**C**) CD86 and (**D**–**F**) CD206 in the ipsilateral brain structures (**A**,**D**) of the frontal cortex (CX), (**B**,**E**) the hippocampus (HIP) and (**C**,**F**) the dorsal striatum (DS). (**G**) Immunoblots representative for the CX, HIP, and DS for CD86 and CD206, together with the total protein amount visualized by the stain-free technique. The order of the samples is the same as in the graphs. *n* = 6, one-way ANOVA, followed by Sidak’s correction for post hoc comparisons; * *p* < 0.05 vs. MCAO or SHAM. Data are presented as the mean ± SEM.

Ischemia significantly increased the expression of Iba1 in the cerebral cortex and in the dorsal striatum (*p* = 0.032 and *p* = 0.0017, respectively), whereas the AP39 treatment reduced the Iba1 level in the frontal cortex in both experimental groups subjected to the SHAM or MCAO procedure (*p* = 0.013 and *p* = 0.0005, respectively) (Figure 5). In the dorsal striatum, in the animals treated with AP39 and subjected to MCAO, a significant reduction in the Iba1 expression was observed (*p* = 0.0002). There were no significant changes in the Iba1 expression in the hippocampus. The MCAO procedure did not evoke any significant changes in the CD86 expression; however, in the group treated with AP39 and subjected to ischemia, a reduced CD86 expression was observed in the frontal cortex and in the dorsal striatum (*p* = 0.048 and *p* = 0.016, respectively) (Figure 6). Both ischemia, as well as the AP39 treatment, did not have any effect on the expression level of CD206 in the analyzed brain structures.

### 2.4. AP39 Modulates the Expression of Neurotrophic Factors and Their Receptors

**Figure 7:**
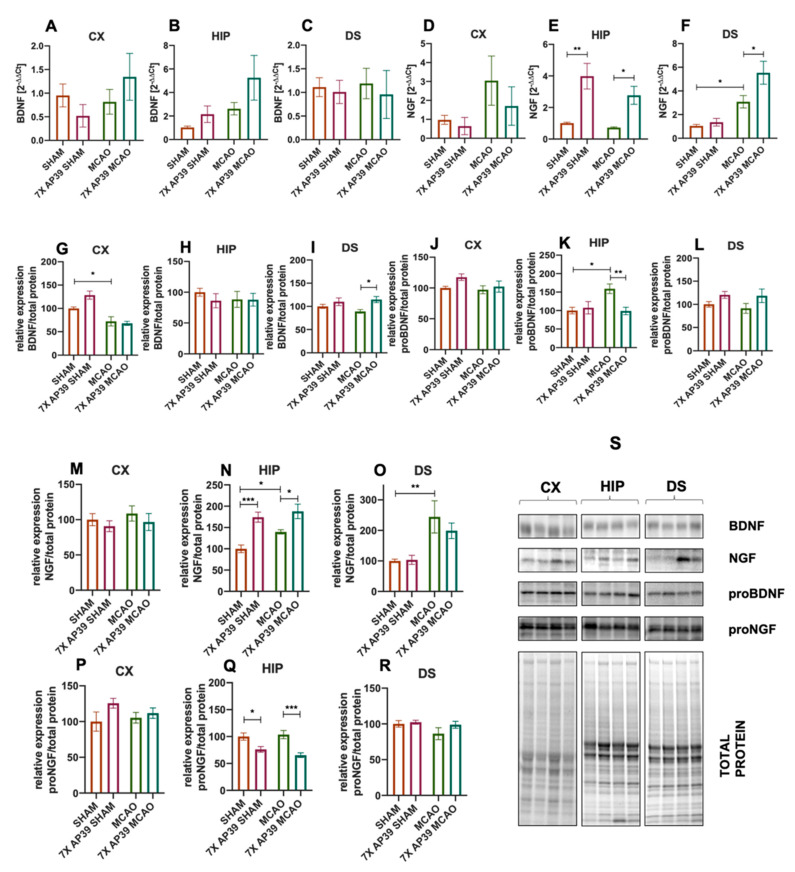
The effect of MCAO and the treatment with AP39 on the mRNA and protein expression levels of the neurotrophic factors. (**A**–**C**) Relative mRNA expression level of BDNF in the frontal cortex (CX), hippocampus (HIP) and in the dorsal striatum (DS), respectively. (**D**–**F**) Relative mRNA expression level of NGF in the CX, HIP and in the DS, respectively. (**G**–**I**) The protein expression level of BDNF in the CX, HIP and in the DS, respectively. (**J**–**L**) The protein expression level of proBDNF in the CX, HIP and in the DS, respectively. (**M**–**O**) The protein expression level of NGF in the CX, HIP and in the DS, respectively. (**P**–**R**) The protein expression level of proNGF in the CX, HIP and in the DS, respectively. (**S**) Immunoblots representative for the CX, HIP and DS for BDNF, NGF, proBDNF and proNGF, together with the total protein amount visualized by the stain-free technique. The order of the samples is the same as in the graphs. *n* = 6, one-way ANOVA, followed by Sidak’s correction for post hoc comparisons; * *p* < 0.05, ** *p* < 0.01 and *** *p* < 0.001 vs. MCAO or SHAM. Data are presented as the mean ± SEM.

Neither the experimental procedure nor the treatment resulted in the changes in *Bdnf* mRNA expression in the analyzed brain structures. MCAO raised the *Ngf* expression level in the dorsal striatum (*p* = 0.038). The AP39 treatment elevated the *Ngf* expression in the hippocampus and in the dorsal striatum of the animals subjected to focal cerebral ischemia (*p* = 0.047, *p* = 0.015, respectively). In the hippocampus in the SHAM-operated animals, the AP39 treatment significantly elevated *Ngf* (*p* = 0.0015) (Figure 7).

The procedure of MCAO elevated the level of NGF in the hippocampus (*p* = 0.048) and in the dorsal striatum (*p* = 0.0023) but had no such effect in the frontal cortex. The effect of the AP39 treatment on the NGF level was observed only in the hippocampus, where in both the SHAM and MCAO-subjected animals, NGF was significantly higher in the AP39-treated rats than in their respective control groups (*p* = 0.0002 and *p* = 0.018, respectively). I/R did not affect the expression of proNGF in any of the brain structures, but the AP39 treatment resulted in a reduced level of proNGF in the hippocampus of both the SHAM and MCAO groups (*p* = 0.026 and *p* = 0.0009, respectively). MCAO resulted in a reduced level of BDNF protein in the frontal cortex but not in the hippocampus or the dorsal striatum, whereas the treatment with AP39 significantly raised the BDNF concentration in the frontal cortex of the 7xAP39 SHAM animals (*p* = 0.025) and in the dorsal striatum of the 7xAP39 MCAO animals (*p* = 0.015). Changes within the level of proBDNF were observed only in the hippocampus, where the MCAO procedure enhanced its expression (*p* = 0.01) but the AP39 treatment prevented this rise (*p* = 0.009) (Figure 7).

**Figure 8:**
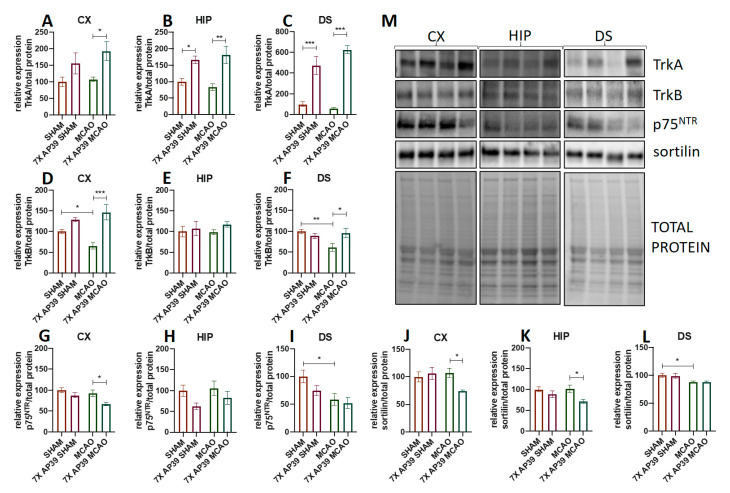
The effect of MCAO and AP39 on the protein expression levels of crucial growth factor receptors. (**A**–**C**) The protein expression level of TrkA in the frontal cortex (CX), the hippocampus (HIP) and in the dorsal striatum (DS), respectively. (**D**–**F**) The protein expression level of TrkB in the CX, HIP and in the DS, respectively. (**G**–**I**) The protein expression level of p75^NTR^ in the CX, HIP and in the DS, respectively. (**J**–**L**) The protein expression level of sortilin in the CX, HIP and in the DS, respectively. (**M**) Immunoblots representative for the CX, HIP and DS for TrkA, TrkB, p75^NTR^ and for sortilin, together with the total protein amount visualized by the stain-free technique. The order of the samples is the same as in the graphs. *n* = 6, one-way ANOVA, followed by Sidak’s correction for post hoc comparisons; * *p* < 0.05, ** *p* < 0.01 and *** *p* < 0.001 vs. MCAO or SHAM. Data are presented as the mean ± SEM.

The MCAO procedure did not change the expression of TrkA in the examined brain structures, but the AP39 treatment significantly elevated the TrkA expression in the frontal cortex of the ischemic animals (*p* = 0.044) and, also, in both the SHAM and MCAO experimental groups in the hippocampus (*p* = 0.0485 and *p* = 0.0017, respectively) and the dorsal striatum (*p* < 0.0001). In the frontal cortex and the dorsal striatum, I/R resulted in a significant decrease in the expression of TrkB (*p* = 0.039 for the frontal cortex (*p* = 0.007 for the dorsal striatum), but there were no significant changes in the hippocampus. In the MCAO animals treated with AP39, the TrkB expression was elevated in the frontal cortex and in the dorsal striatum. The MCAO procedure caused a significant decrease of p75^NTR^ in the dorsal striatum only (*p* = 0.027), whereas, in the animals subjected to ischemia and treated with a H_2_S donor, a reduced expression of this receptor in the frontal cortex was observed (*p* = 0.041). MCAO reduced the sortilin expression in the dorsal striatum (*p* = 0.039), whereas the AP39 treatment reduced the expression of sortilin in the frontal cortex and in the hippocampus of the animals subjected to ischemia (*p* = 0.049 and *p* = 0.016) (Figure 8).

### 2.5. AP39 Mitigated MCAO-Induced Caspase 3 Activation

**Figure 9:**
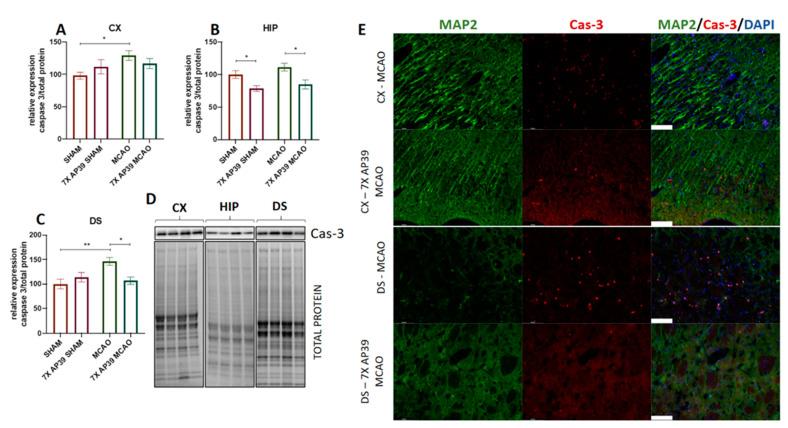
The effect of MCAO and AP39 on the level of the cleaved form of caspase 3 (**A**) in the frontal cortex (CX), (**B**) in the hippocampus (HIP) and (**C**) in the dorsal striatum (DS). (**D**) Immunoblots representative for the CX, HIP and DS for cleaved caspase 3, together with the total protein amount visualized by the stain-free technique. The order of samples is the same as in the graphs. *n* = 6, one-way ANOVA, followed by Sidak’s correction for post hoc comparisons; * *p* < 0.05, ** *p* < 0.01 vs. MCAO or SHAM. Data are presented as the mean ± SEM. (**E**) Representative photographs of brain sections double-immunostained against the neuronal marker, microtubule associated protein 2 (MA2, green) and cleaved caspase 3 (Cas-3, red). Stained sections were mounted with a DAPI-containing medium for nuclei staining (blue). Photographs are focused on the motor cortex and the dorsal striatum. Photographs for the hippocampus are not shown, since there were no clear changes between SHAM and MCAO on the fluorescence level. Scale bar = 100 µm.

The analysis of the active form of caspase 3 was performed using the immunoblot and brain sections double-staining immunofluorescence assay. In the immunoblot assay, MCAO resulted in a significant elevation of cleaved caspase 3 expression in the frontal cortex (*p* = 0.031) and in the dorsal striatum (*p* = 0.0048) (Figure 9). In the experimental group pretreated with AP39, a significant decrease was observed in the dorsal striatum and in the hippocampus of the animals subjected to the I/R model (*p* = 0.016 and *p* = 0.018, respectively). In the SHAM-operated animals pretreated with AP39, a decrease of cleaved caspase 3 was observed only in the hippocampus (*p* = 0.044). Immunofluorescence staining for cleaved caspase 3 revealed that the treatment with AP39 locally reduced the level of active caspase 3 in the neurons of the frontal cortex and the dorsal striatum of the animals subjected to focal cerebral ischemia (Figure 9).

## 3. Discussion

AP39 (50 nmol/kg) administration revealed the neuroprotective activity of this compound and the notably reduced infarct volume and neurological deficit in the experimental groups treated with AP39 and subjected to MCAO. These suggest that mitochondrial H_2_S might evoke some adaptative changes in the brain tissue. In the dorsal striatum, the most damaged brain area after MCAO [49], the concentration of free H_2_S was significantly decreased 24 h after I/R (Figure 2), and this is in agreement with other reports [29]. AP39 preserved the level of free H_2_S at the basal level but increased the bound forms of H_2_S (i.e., persulfides or polysulfides), frequently called physiologically available H_2_S. AP39 increased the bound and available pool of H_2_S also in the frontal cortex and in the hippocampus and markedly reduced the infarct volume and neurological deficit (Figure 1). It is well-established that mitochondria metabolize sulfide for respiration [8], and since AP39 delivers H_2_S in the mitochondria [39,50], it is probable that the observed neuroprotection comes from increased catabolism of H_2_S by SQOR, which provides electrons to mitochondrial chain complex III via coenzyme Q and enhances the ATP production and rescues the mitochondrial membrane potential [9,11,51,52]. H_2_S-mediated persulfidation of the proteome may change the functions of specific proteins; thus, the pattern of persulfidation and the increased level of bounded H_2_S might be a potential mechanism of H_2_S-mediated brain preconditioning. Indeed, AP39 has previously been shown to persulfidate mitochondrial proteins [53,54]. This theory might be supported by the recent discovery where persulfidation of the proteome by means of H_2_S is mainly involved in primary metabolic pathways such as glycolysis and the Krebs cycle [55]. These pathways integrate crucial reactions, maintaining an energetic balance in cells called homeostasis, which is disturbed just after ischemia onset. The reaction of persulfidation, which may change the functions of crucial proteins or enhance oxidative phosphorylation, is dependent on SQOR; however, the expression/activity of this enzyme is very low in the brain [56]. Recent data indicate that the preconditioning of animals with H_2_S inhalation induced brain tolerance to ischemia and that the mechanism of preconditioning relied on the upregulation of SQOR [8]. However, to verify whether such an effect is also crucial in the mechanism of action of the slow-releasing and targeted compound AP39 demands further and more detailed studies.

Following brain ischemia, microglial and astroglial cells are activated within hours. This leads to the production of numerous proinflammatory cytokines and chemokines. This evokes acute neuroinflammation, which promotes further brain cell damage. The functions of activated microglia during ischemic stroke are not completely understood, and their distinct roles may result from the different microglia phenotypes. In our current study, I/R resulted in a microglia activation, measured by Iba1 expression, in the brain areas specifically prone to the infarction—the frontal cortex and the dorsal striatum. The AP39 treatment reversed this effect, securing Iba1 expression at the basal level. In the frontal cortex and in the dorsal striatum of the animals treated with AP39 and subjected to ischemia, we observed a reduced expression of CD86, a marker of the cytotoxic M1-like phenotype of the microglia. This may suggest that AP39 reduced the microglia polarization towards the cytotoxic M1-like phenotype, but on the other hand, we did not notice an elevated level of CD206 (a marker of neuroprotective M2-like phenotype of the microglia) expression in any experimental group. M1-like microglia secrete proinflammatory cytokines, such as TNFα, Il-1β, Il-12, Il-23 and nitrogen monoxide (NO); as such, they exacerbate inflammation and tissue injury. In contrast, M2-like microglia secrete anti-inflammatory cytokines (TGF-β, IL-4, IL-10 and Il-13), and these cells suppress inflammation and promote tissue recovery [57,58]. In this study, the microglia activation results were in line with the data on the proinflammatory cytokine levels. AP39 profoundly reduced the mRNA and protein levels of Il-1β, Il-6 and TNFα, which are normally highly elevated after MCAO. In the other reference strategies of brain preconditioning, such as by ischemic preconditioning, 3NP preconditioning or remote preconditioning, similar effects were observed [59,60]. The AP39 treatment reduced the levels of the cytotoxic mediators of acute neuroinflammation: Il-1β, Il-6 and TNFα (mRNA and protein) but also modulated the release of the survival factor, Il-10. In the dorsal stratum, the brain structure mostly affected by I/R, the mRNA and the protein levels of Il-10 were significantly elevated after the treatment with AP39 in both groups that were subjected to MCAO and SHAM. This may suggest that the H_2_S donor administered before ischemia induced some adaptative changes and, as a result, increased the release of anti-inflammatory Il-10, which mediated its neuroprotective effect through activation of the JAK1-STAT3 signaling pathway [61,62]. After ischemia onset, one of the main mechanisms activating glial cells, is dependent on the molecules released by dying neurons [63,64]. On the other hand, an enhanced H_2_S catabolism that might occur after the AP39 treatment could promote survival of neurons and, thus, could indirectly reduce the activation of glial cells. There are data that H_2_S is capable of directly silencing microglial cell activation by inhibition of the p38-MAPK signaling pathway [65]. However, whether neuroprotection is caused by reduced microglia activation due to the direct influence of AP39 on these cells or it is a result of reduced neuronal death that might be metabolically enhanced by AP39 or both mechanisms are engaged is unclear, and this issue demands further and detailed studies.

Neurotrophins such as BDNF or NGF are considered as mediators of neuronal and synaptic plasticity. In our study, I/R reduced the BDNF signaling pathway in the dorsal striatum, where the expression of TrkB, sortilin and the p75^NTR^ receptor was reduced; however, the level of BDNF (protein and mRNA) was unchanged, whereas proBDNF was upregulated in the hippocampus only. Since the dorsal striatum is, in the MCAO model, the core of ischemia, where extended necrotic lesions with enhanced proteolytic activity are formed, it is possible that the observed changes are due to cellular loss. The treatment with AP39 significantly raised the expression of the TrkB receptors in the frontal cortex and in the dorsal striatum, decreased the expression of p75^NTR^ receptor in the frontal cortex and decreased the expression of sortilin in the hippocampus. BDNF, acting through TrkB, promotes cell survival and synaptic plasticity. Unmature proBDNF, by interacting with the p75^NTR^ receptor and a coreceptor sortilin, promotes cell death and degradation of the synapses [66,67,68]. Thus, despite the lack of significant differences in the level of BDNF (protein and mRNA), the neuroprotective activity of the BDNF-TrkB pathway seems to be enhanced, and the proapoptotic effect dependent on the signaling through p75^NTR^-sortilin might be reduced in AP39-treated rats. In a similar way, the H_2_S donor NaSH was reported to reveal neuroprotective activity in in vitro studies [69]; however, to our knowledge, there are no in vivo data on such mechanisms in an animal model of stroke. 

In the case of NGF, the obtained results indicated that the stroke itself triggers the endogenous protective mechanism related to the action of this factor. The increase in the level of NGF mRNA in the frontal cortex and dorsal striatum and upregulation of the NGF protein in the dorsal striatum with no change in the level of TrkA suggests an intensification of the NGF-TrkA protective signaling pathway by stroke. In turn, the reduced level of the p75NTR receptor and its coreceptor sortilin, even with no changes in proNGF, means that the cytotoxic effect of proNGF might be slightly reduced.

AP39 evoked the strongest protective effect on the hippocampus, where the expression of NGF (mRNA and protein) was raised and the expression of proNGF in both experimental groups (7xAP39 SHAM and 7xAP39 MCAO) was decreased. Moreover, AP39 significantly increased the TrkA expression in both experimental groups in the hippocampus and in the dorsal striatum, whereas, in the frontal cortex, a rise in TrkA was observed only in the animals subjected to ischemia. Sortilin was downregulated in the hippocampus of the animals treated with AP39 and subjected to ischemia. NGF promotes cell survival and synaptic plasticity by activation of the TrkA receptor [70,71]. Similar to proBDNF, proNGF, by interacting with the p75^NTR^ receptor, promotes cell death and the degradation of synapses [66,67,68]. Sortilin is considered as an essential coreceptor necessary for proNGF-induced neuronal cell death [72]. BDNF and NGF stimulate dendrites and axon outgrowths, the formation of new synaptic connections [73,74]. Thus, neurotrophins play a crucial role in the poststroke recovery of brain tissue, especially within the periinfarct area, where neuronal pathways are physically interrupted by the core of ischemia. In this study, AP39 enhanced the neurotrophic signaling of both factors, reducing the opposite effect of the pro-forms. An observed brain tolerance to ischemia after AP39 treatment suggests that this H_2_S donor may reveal its effect by enhancing the signaling pathways of the neurotrophic factors. However, this requires further and detailed studies. 

In our study, MCAO increased the expression of cleaved caspase 3 in the frontal cortex and in the dorsal striatum, suggesting the induction of apoptosis. However, AP39 reduced the expression of caspase 3 in the hippocampus in both the experimental groups and in the dorsal striatum in animals subjected to focal cerebral ischemia. These changes were observed both in the immunoblot, as well as in the immunofluorescent staining of the brain sections. Both the dorsal striatum and, locally, the frontal cortex are brain structures that were highly damaged 24 h after MCAO [75]. MCAO did not increase the activation of caspase 3 in the hippocampus at 24 h postischemia; however, caspase 3 is an executive enzyme of apoptosis, which is considered a delayed and regulated mechanism of cell death. In the hippocampus, the granule cells of the CA1 layer are the most vulnerable to damage; however, the peak of apoptotic degradation of this cell population occurs days after such changes are observed in the cortex or the dorsal striatum [46,76]. In AP39-treated animals, the decrease of caspase 3 activation in the dorsal striatum might be related with the secured concentration of free H_2_S and the elevated level of bound H_2_S. The increased oxidation of H_2_S enhances the functions of the respiratory chain within mitochondria. The increased catabolism of H_2_S not only is the source of ATP but also provides mitochondrial membrane potential, which prevents the cytochrome c-mediated initiation of apoptosis. Moreover, in the hippocampus of the ischemic groups, as well as in the AP39 pretreated groups, the downregulation of caspase 3 activation may arise from the enhanced signaling of NGF-TrkA, and this is consistent with other data indicating the protective effects of NGF during a stroke [70,71].

It is prudent to point out the limitations in this study. Although our study clearly demonstrated that AP39 (50 nmol/kg) could prevent detrimental signaling in the brain and subsequent brain injury during MCAO stroke, we only evaluated one dose of AP39 and one administration route. An oral route would be the most clinically viable, although, in an acute setting, intravenous administration is warranted. However, the ADME/PK properties of AP39 are not known, and extensive work is required to optimize the dose response, formulation and delivery. We did not show a dose response in our current study, and this needs to be looked at in future studies. Similarly, we dosed prophylactically (i.e., prior to MCAO) rather than therapeutically. Although there is considerable merit in preventative treatments for stroke, therapeutic dosing (i.e., after MCAO) is needed for future studies. Another limitation of this study is that we only used male rats, whereas stroke aspects in males and females might be different. Studies on female animals are much more challenging due to the necessity of estrus cycle monitoring, which hugely increases the number of animals for each experimental group. The estrus cycle has direct influence on stroke outcomes. Nevertheless, this was the first study on the possible beneficial role of AP39 in brain ischemia. Our aim was to provide data that might be easily compared to the other disease models, such as myocardial infarction or acute lung injury studies where male rats were used in experiments. Altogether, compounds like AP39 need to be examined in both sexes as proof of principle before large-scale studies are carried out. Recent data indicate that preconditioning with H_2_S significantly increases the catabolism of H_2_S through SQOR, which stimulates mitochondrial functions [8]. This suggests that the repeated administration of AP39 before ischemia onset might similarly enhance the SQOR expression, which, in turn, might elevate the level of bound H_2_S and, thus, enhance the functions of mitochondria and rescue neurons in the ischemic area. What is more, modified, persulfidated proteins may change their functions, but it is unknown which proteins are being persulfidated due to H_2_S catabolism. It is known that AP39 increases complex II and III activity and raises persulfidation; however, this research unfortunately does not explain these urgent issues in the model of cerebral ischemia [11,54]. As such, to confirm such a breakthrough mechanism, more detailed studies concerning the mitochondria metabolism and, also, the global protein persulfidation patterns are necessary. We are currently determining the additional parameters to confirm the mechanism of action, e.g., therapeutic effects on cellular bioenergetics and persulfidome analysis after therapeutic treatment.

## 4. Materials and Methods

### 4.1. Animals and Experimental Design

All experiments were performed on male Sprague-Dawley rats (280–320 g, Charles Rivers). The animals were maintained on a normal day–night cycle at 22 ± 2 °C with free access to food and water. All experimental protocols were in accordance with the Guide for the Care and Use of Laboratory Animals published by the National Institutes of Health and were approved by the Second Local Ethical Committee at Institute of Pharmacology Polish Academy of Sciences on 19 January 2017 (project identification code: 11/2017). All studies involving animals were reported according to the ARRIVE (Animal Research: Reporting of In Vivo Experiments) guidelines, including the procedure for blinding the investigators to the identities of the animals at each point of the experiment.

### 4.2. Animal Treatment

Animals were randomly allocated into the following groups: SHAM, MCAO, 7xAP39 SHAM and 7xAP39 MCAO. For each group, *n* = 6–8 animals. In the 7xAP39 SHAM and 7xAP39 MCAO groups, a 7-day treatment with AP39 (50-nmol/kg b.w., i.v.) with the last dose 72 h before the induction of ischemia was performed. The time lapse between the onset of reperfusion and animal decapitation and tissue collection was 24 h. AP39 was dissolved in 1% solution of DMSO in PBS. In the control groups, which were MCAO and SHAM, 1% solution of DMSO in PBS was administered in the same injection scheme as in the AP39 pretreated groups. AP39 was synthesized as previously described by us [39].

### 4.3. Focal Cerebral Ischemia Model

MCAO was elicited according to the method of Longa et al. to induce transient focal cerebral ischemia as previously described [42,43,77]. All surgical procedures were carried out under a stereoscopic microscope (Leica, A60F, Wetzlar, Germany), and body temperature was maintained at a physiological level using a heating blanket (homeothermic blanket system; Harvard Apparatus). Arterial occlusion was confirmed using a blood flowmeter (PeriFlux System 6000, Perimed, Jakobsberg, Sweden), and a 70% blood flow reduction (or higher) was considered to indicate a successful procedure. The rats were anesthetized with 5% isoflurane for induction and 2.5% isoflurane for maintenance. After exposure of the left external carotid artery (ECA), the internal carotid artery (ICA) and the common carotid artery (CCA), all branches of the ECA were coagulated, and the artery was secured. The ICA and CCA were temporarily secured with microvascular clips. A silicone-coated filament (Doccol, Sharon, MA, USA) was introduced into the lumen of the ECA and advanced until the blood flow decreased. The clip was removed from the CCA, and after the administration of local anesthesia with 0.2% ropivacaine (AstraZeneca, Wedel, Germany), the wound was secured with silk sutures. The occlusion was maintained for 90 min. Afterwards, the wound was reopened, and the filament was removed to restore blood flow. The wound was closed with sutures. The SHAM operation was carried out as described above without insertion of the filament. Twenty-four hours after the procedure, the neurological deficit was assessed for each animal, and next, the animals were sacrificed, and the brain tissue was dissected appropriately for the assay.

### 4.4. Neurological Deficit

The neurological deficit was assessed 24 h after reperfusion. The 10-point grading system of Philips et al. 2000 was used, as we described previously [78]. Briefly: animals were subjected to assessment at 1 h and 24 h after MCAO. The following neurological symptoms were assessed and scored: 4 points were granted when animals pushed in a contralateral direction did not reveal any resistance, 3 points were granted when animals were circling in a contralateral direction, if animal revealed contralateral shoulder adduction, 2 points were added and 1 point was granted if contralateral forelimb flexion was observed. Zero points indicated no neurological deficit, whereas 10 points was the maximal neurological deficit. 

### 4.5. TTC Staining—Measurement of the Infarct Volume

For determining the volume of the infarct, separate experimental groups were prepared (for each group, *n* = 6). Twenty-four hours after MCAO, the animals were decapitated, and their brains were immediately removed and sliced using a brain matrix (Harvard Apparatus, Holliston, MA, USA). Coronal sections (2 mm thick) were stained with a 1% solution of 2,3,5-triphenyltetrazolium chloride (TTC) dye (Sigma Aldrich, St. Louis, MO, USA) in 0.01-M phosphate-buffered saline (Sigma Aldrich, St. Louis, MO, USA) at 37 °C for 10 min in the dark. Next, the sections were fixed in 10% phosphate-buffered formalin (Sigma Aldrich, St. Louis, MO, USA) for 30 min at 4 °C. The stained and fixed brain slices were photographed using a surgical microscope equipped with a camera (Moticam, Motic, Germany) by an investigator blinded to the subject’s identity. The infarct volume was determined by NIH ImageJ software (National Institutes of Health, version 8.0) by the same investigator. The infarct volume was calculated as a sum of each outlined white area multiplied by the thickness of the brain section and was expressed in mm^3^.

### 4.6. Luminex Assay

Twenty-four hours after surgery, the brains were removed from the skull, and the ipsilateral hemispheres were immediately dissected on ice from the frontal cortex, the hippocampus and the dorsal striatum and then stored at −80 °C. Next, the frozen tissue was weighed, and 10% (*w*/*v*) homogenates were prepared by homogenization in 0.01-M phosphate buffer, pH 7.4 using an IKA-ULTRA-TURRAX T8 homogenizer. Obtained homogenates were centrifuged at 10,000× *g*, and the supernatants were analyzed for the levels of Il-1β, Il-6, Il-10 and TNFα using a commercially available Milliplex^TM^ MAP Kit (Millipore, Billerica, MA, USA), according to the manufacturer’s protocols. Briefly, the homogenates were incubated with a mixture of target-specific antibody-immobilized magnetic beads on a sealed 96-well microplate for 2 h at RT with agitation on a plate shaker. Next, using a handheld magnet, the magnetic beads were washed, using a provided washing buffer, three times. The detection antibodies were added, and the sealed plate was incubated for 1 h at RT with agitation. Next, streptavidin-phycoerythrin solution was supplied to each well, containing standards, quality controls and samples (in triplicates), and the plate was incubated for a further 30 min at RT with agitation. Then, using a handheld magnet, the plate was washed three times, and after gentle removing of the well contents, DriveFluid was added, and the magnetic beads were resuspended on a plate shaker for 5 min. The quantitative analysis was performed using a MAGPIX Luminex analyzer with xPONENT software (Luminex Corporation, Austin, TX, USA). The levels of the analytes were calculated based on standard curves using the spline curve-fitting method and were expressed in pg/mL of homogenate.

### 4.7. Western Blot

The tissues of the frontal cortex, hippocampus and dorsal striatum were homogenized in 2% SDS containing 1-mM PMSF, 1-mM Na_2_VO_4_, 20-mM NaF and a mixture of phosphatase-proteinase inhibitors (Sigma Aldrich) using Ultra-Turrax and ultrasonic homogenizers. After denaturation at 95 °C for 10 min, insoluble debris were removed by centrifugation at 10,000× *g* for 10 min at 4 °C. The protein concentration in the supernatants was determined using the BCA protein assay kit (Thermo Scientific, Waltham, MA, USA). After setting a proper protein concentration, the solutions were mixed with loading buffer (containing 10% 2-mercaptoethanol) at a ratio of 1:1 and heated for 10 min at 95 °C. The samples were loaded on a gradient of 8–16% SDS polyacrylamide gels (Criterion, TGX-Stain-free gel, Bio-Rad, Hercules, CA, USA) at a total protein concentration of 30 µg/10 µL, and electrophoreses (200 V, 45 min) was performed. Next, the proteins were semi-dried and transferred (TurboBlot, Bio-Rad) to PVDF membranes and were blocked in 5% solution of albumin. The membranes were incubated overnight at 4 °C with primary antibodies at the appropriate concentration (Table 1). After an overnight incubation, the membranes were washed in TBST and then in 1% albumin solution and were incubated with their respective secondary antibodies conjugated with peroxidase in 1% albumin solution for 1 h at RT. After washing, the membranes were developed using the ECL method (i.e., Western Bright Quantum, Advansta Inc., San Jose, CA, USA). The chemiluminescence of the membranes was imaged with the G-Box Imaging System (Syngene, Frederick, MD, USA), and the protein expression was analyzed with Gene Tools software (Syngene, Frederick, MD, USA) and expressed relative to the total protein content in the sample.

### 4.8. Real-Time qPCR

The rats were decapitated 24 h after reperfusion; the brains were immediately removed, and the selected brain structures: the frontal cortex, hippocampus and dorsal striatum were isolated and immersed in Fix-RNA stabilization solution (Eurx, Poland) for 24 h at 4 °C to preserve the RNA. The brain structures were homogenized in Extracol (Eurx, Poland) with a bead homogenizer (Bead Ruptor 24 Elite, Omni International), and the total RNA from the obtained homogenates was isolated and purified using the Gene Matrix Universal Purification kit (Eurx, Poland) according to the manufacturer’s protocols. The integration, as well as the concentration, of isolated was verified, and the isolated RNA was stored at −80 °C until used. Reverse transcription reactions and real-time PCR were performed using the CFX Connect real-time System (Bio-Rad, Hercules, CA, USA). The total RNA was transcribed into cDNA using the smart First Strand cDNA synthesis kit (Eurx, Poland) according to the manufacturer’s procedure. The obtained cDNA was stored at −80 °C until used. The relative expressions of selected genes were determined using the commercially available TaqMan Gene Expression Assays (Applied Biosystems, Waltham, MA, USA) according to the manufacturer protocols (Table 2). Real-time PCR was performed using the Pro qPCR master mix (Eurx, Poland) according to the manufacturer’s procedure. The expression levels of each gene were normalized to the reference genes levels; the fold change in the expression was determined using the ΔΔc(t) method of relative quantification. 

### 4.9. Tissue Fixation for the Immunofluorescent Double-Staining

The groups of animals dedicated for immunofluorescent staining were subjected to an intracardiac 4% paraformaldehyde (PFA) perfusion 24 h after the surgical procedure. First, the animals were deeply anesthetized with ketamine (80 mg/kg) and xylazin (20 mg/kg). Then, rats were transcardially perfused with 250 mL of NaCl solution (0.9%; 32 °C) until all the remaining blood was removed. Next, the animals were perfused with 500 mL of 4% PFA in a 0.1-M PBS. After the procedure, the brains were removed and kept in the same 4% PFA solution overnight. Afterwards, the brains were transferred to a 10% sucrose solution containing a 0.1% sodium azide solution in 0.1-M PBS and stored for 24 h. Next, the brains were transferred to a 20% and, then, to a 30% sucrose solution with 0.1% sodium azide in 0.1-M PBS and stored until it sunk each time. Next, the brains were cut into coronal 10-μm sections using an automatic cryotome (Leica CM 1860). The sections were attached to SuperFrost (Thermo Scientific, Waltham, MA, USA) microscopy slides and stored at −20 °C until stained and analyzed. 

### 4.10. Immunofluorescent Double-Staining

Before the procedure of staining for caspase 3, a heat-induced antigen retrieval with trisodium citrate (pH = 9) was performed. The sections were washed twice in TBS and permeabilized in 0.3% Triton X-100 in TBS for 30 min on a shaker at RT. Afterwards, an unspecific binding of the antibodies was blocked using 10% normal serum in 0.1% Triton X-100 in TBS for one hour at RT. Next, after removing the blocking solution, the sections were incubated with specific primary antibodies (Table 1, anti-caspase 3 and anti-MAP-2, a neuronal marker) and dissolved in 2% normal serum in 0.1% Triton X-100 in TBS on a shaker at 2–8 °C overnight. The next day, the slides were incubated for one hour on a shaker at room temperature. Then, the antibody solution was removed, and the slides were washed twice in 2% specific normal serum in 0.1% Triton X-100 in TBS. Afterwards, a mixture of specific secondary antibodies conjugated with FITC or TexasRed dissolved in 0.1% Triton X-100 in TBS was added to the sections and incubated for 3 h in the dark on a shaker at RT. Following this, the slides were washed twice in 0.1% Triton X-100 in TBS. Next, the slides were mounted in Fluoroshield Mounting Medium with DAPI (Sigma-Aldrich), and by using a specific channel, the slides were scanned using a Leica DMI8 fluorescent inverted microscope. The localization of the active form of caspase 3 was captured using a Leica DFC450C camera and Leica LAS X software.

### 4.11. Measurement of H_2_S Content

**Figure 10:**
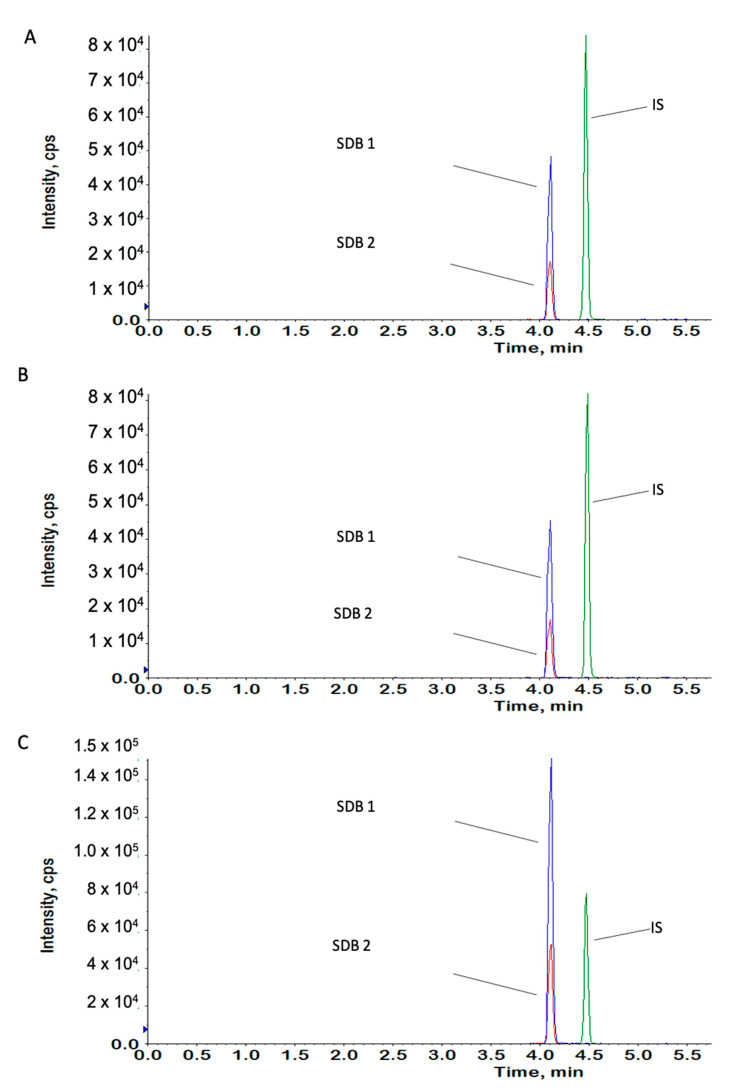
Analysis of hydrogen sulfide (H_2_S) in rat brain structures using LC-MS/MS. Chromatograms obtained from (**A**) blank rat brain homogenate, spiked with SDB, at a concentration of 800 nM, (**B**) hippocampus homogenate of a rat from the SHAM group—calculated H_2_S concentration = 844 nM and (**C**) the same hippocampus homogenate of a rat from the SHAM group pretreated with DTT—calculated H_2_S concentration = 2600 nM. SDB 1: *m*/*z* = 415/193 and SDB 2: *m*/*z* = 415/223.

Bioavailable biochemical forms of H_2_S, including free and dithiothreitol (DTT)-released forms (e.g., persulfides), were measured in the rat brain structures according to the previously described monobromobimane (MBB) method with some modifications [79]. Briefly, the rat brain structures were homogenized (Bead Ruptor 24 Elite, Omni International) with ice-cold Tris-HCl (100 mM; pH = 8.5) at a ratio of 1:9 (*w*/*v*), followed by centrifugation at 12,000× *g* for 20 min. The supernatant was frozen at −80 °C until the next day. To determine the free and DTT-released H_2_S, the supernatants were thawed at RT for 10 min and then pipetted into two amber tubes (at the volume of 30 µL each). To the first tube, 10 μL of 10-mM DTT solution was added, and the sample was incubated at 37 °C for 15 min on the thermostatic block with gentle shaking. To the second tube, 10 μL of distilled water was added, and the sample was placed on ice for 15 min. Then, 95 μL of a derivatization reagent mixture (20-mM MBB in acetonitrile:2-mg/mL EDTA:100-mM TRIS-HCl (pH = 8.5), 35:10:50, *v:v:v*) was added to both samples. After shacking on a block heater for 60 min, the reaction was stopped by adding 50 μL of ice-cold acetonitrile containing 10% formic acid, and the samples were placed on ice for 5 min. The derivatization process was carried out in the dark at 20 °C, according to the previous recommendations [80]. During the last step of sample preparation, 20 μL of 1-μg/mL valsartan solution (internal standard—IS) was added, and after centrifugation at 12,000× *g*, 4 °C for 10 min, 1 μL of the supernatant was injected into the LC-MS/MS system (Exion LC AC HPLC coupled to a Sciex QTRAP 4500 triple-quadrupole mass spectrometer, both from Danaher Corporation, Washington, DC, USA). The chromatographic separation of sulfide dibimane (SDB; a product of H_2_S derivatization) was performed on the Hypersil Gold^TM^ C18 analytical column (2.1 × 50 mm, 3 µm; Thermo Scientific, USA) with a gradient elution using a mobile phase containing 0.1% (*v*/*v*) of formic acid in acetonitrile and in water. Electrospray ionization (ESI) in the positive ion mode was used for ion production. The mass spectrometer was operated at unit resolution in the selected reaction monitoring mode, monitoring the transition of the protonated molecular ions *m*/*z* 415–193 (CE = 25 eV) and *m*/*z* 415–223 (CE = 31 eV) for SDB (the first pair was used as a quantifier and the second for the identity verification qualifier) and *m*/*z* 436–235 (CE = 42 eV) for the IS. The ion source temperature was maintained at 500 °C, and the ion spray voltage was set at 5500 V. The curtain gas (CUR) was set at 40 and the collision gas (CAD) at medium. The calibration curve was prepared by spiking 25 μL of brain homogenate supernatant with 5 μL of the standard working solution of SDB (synthetized in-house according to Wintner et al. [81]) to yield final concentrations of 50, 100, 200, 400, 800, 1600 and 3200 nM, and after adding 10 μL of H_2_O, 95 μL of the reagent mixture without MBB (acetonitrile:2-mg/mL EDTA:100-mM TRIS-HCl (pH = 8.5), 35:10:50, *v:v:v*), 50 μL of ice-cold acetonitrile containing 10% formic acid and 20 μL of the IS, the samples was centrifuged, and the supernatant was injected into the LC-MS/MS system. Data acquisition and processing were accomplished using Analyst version 1.7 software. The calibration curve in the range from 50 to 3200 nM was constructed by plotting the ratio of the peak area of the SDB to the IS versus the SDB concentration and generated by a weighted (1/x·x) linear regression analysis (Figure 10). The concentrations detected in the rat brain structures were expressed in nM. The concentrations of DTT-released H_2_S were calculated by subtracting the sum of the basal SDB concentration (derivatization of the DTT solution in TRIS-HCl) and concentration of the free H_2_S from the total amount of H_2_S determined in the corresponding sample pretreated with DTT.

### 4.12. Statistics

All data are expressed as the means ± SEM. The infarct volume, protein and mRNA expression and the level of H_2_S were analyzed using one-way ANOVA. If statistical significance was found through an analysis using an ANOVA, Sidak’s post hoc test was then conducted to test the comparisons. A Mann–Whitney *U* test was applied for the analysis of the neurological deficits. *p* < 0.05 was considered statistically significant. The statistical analyses were performed using GraphPad Prism version 8.01 software for a Mac (GraphPad, San Diego, CA, USA).

## 5. Conclusions

In conclusion, our study confirms the beneficial effect of AP39 and mitochondria-targeted sulfide approaches in an animal model of focal cerebral ischemia. The investigation suggests an anti-inflammatory activity of AP39 and modulatory effect towards neurotrophic factors and their signaling pathways. The enhancement of the neurotrophic signaling pathway could result in a reduced caspase 3 activation and subsequent reduction in cellular damage. The repeated administration of low doses of a slow-releasing and mitochondria-targeted H_2_S donor preserved the free H_2_S at the basal level 24 h after ischemia onset and increased the bound fraction of H_2_S, which was composed inter alia of persulfidated proteins. 

## Figures and Tables

**Table 1:** The list of primary and secondary antibodies used in Western blot and fluorescence microscopy techniques.

Antibody	Manufacturer	Catalog Number	Dilution Used
pro-BDNF	Alomone	AGP-032	1:300
mBDNF	Abcam	ab108319	1:1000
pro-NGF	Alomone	AGP-031	1:300
NGF	Alomone	AN-240	1:300
TrkA	Alomone	ANT-018	1:300
TrkB	Alomone	ANT-019	1:400
p75^NTR^	Alomone	ANT-007	1:300
Sortilin	Alomone	ANT-009	1:500
Iba1	Abcam	ab5076	1:1000
Caspase 3	Cell Signaling	14220S	1:1000
CD206	Abcam	ab125028	1:1000
CD86	Santa Cruz Biotechnology	sc-28347	1:300
MAP2 (neuronal marker)	Abcam	ab5392	1:1000
goat anti-guinea pig (HRP)	Abcam	ab97155	1:10,000
goat anti-rabbit (HRP)	Abcam	ab6721	1:5000
donkey anti-goat (HRP)	Abcam	ab97120	1:10,000
goat anti-chicken (Alexa Fluor 488)	Abcam	ab150173	1:300
donkey anti-rabbit (Texas Red)	Abcam	ab6800	1:1000

**Table 2:** TaqMan expression assays used for RT-PCR experiments, including house-keeping genes (HKG) used for the results normalization.

Gene	Assay ID:
*Ngf*	Rn01533872_m1
*Bdnf*	Rn01484924_m1
*Il-6*	Rn00561420_m1
*Il-1ß*	Rn00580432_m1
*T* *nfa*	Rn99999017_m1
*Il-10*	Rn99999012_m1
*Ppia*—HKG	Rn00690933_m1
*Ywhaz*—HKG	Rn00755072_m1
*Hprt1*—HKG	Rn01527840_m1
